# Supplementary material for: Loose Morphology and High Dynamism of OSER Structures Induced by the Membrane Domain of HMG-CoA Reductase
Source: Int J Mol Sci. 2021 Aug 24;22(17):9132. doi: 10.3390/ijms22179132 (PMC8430881; doi:10.3390/ijms22179132)
Supplement: Supplementary file 1 [file ijms-22-09132-s001.zip › Grados 2021 - Movie legends.pdf]

International Journal of *Molecular Sciences*

MDPI

SUPPLEMENTARY MATERIALS

Movie 1: Root epidermis from *Arabidopsis* 1S:GFP transgenic plant

Life imaging of root epidermis from 9-day old *Arabidopsis* 1S:GFP seedling. A panoramic video was captured by confocal laser microscopy at a fixed Z-position, at times indicated at the lower left corner. Twenty frames were taken every 3.3 sec and are shown at 5 frames per sec (17 times acceleration). Big *ER-HMGR domains* show for and back motion, whereas the ER network and small *ER-HMGR domains* are highly dynamic. In the upper left corner, a nuclear *ER-HMGR domain* separates briefly from the nuclear envelope. The movement of big *ER-HMGR domains* is also visible in the bright field channel. Iterative reproduction allows a better comparison of dynamic and static structures. Scale bar, 10 µm.

Movie 2: Dynamism of the *ER-HMGR domains* and ER network

Life imaging of root epidermal cell from *Arabidopsis* 1S:GFP transgenic seedling. The video was captured by confocal laser microscopy at a fixed Z-position, at times indicated at the lower left corner. Twenty frames were taken every 3.3 sec and are shown at 4 frames per sec (13 times acceleration). On the upper left corner, the big *ER-HMGR domain* shows for and back motion, blurry fluctuating borders, and dynamic connections to the ER network. The small *ER-HMGR domains* are highly dynamic. They move along ER strands or together with the network. The movement of big *ER-HMGR domains* is also visible in the bright field channel. Iterative reproduction allows a better comparison of dynamic and static structures. Scale bar, 5 µm.

Movie 3: Nuclear *ER-HMGR domains*

Life imaging of two nuclei from adjacent root epidermal cells in *Arabidopsis* 1S:GFP seedling. The video was captured at a fixed Z-position by confocal laser microscopy, at times indicated at the lower left corner. Twenty frames were taken every 3.3 sec and are shown at 5 frames per sec (17 times acceleration). The two nuclear *ER-HMGR domains* have spherical-ovoid moving shapes and blurry borders. The fluctuating foggy appearance on the OSER surface may reflect incorporation or emergence of ER membrane material. The dynamism of *ER-HMGR domains* is also visible in the bright field channel. Scale bar, 5 µm.
